# Asymmetric Cell Division and Notch Signaling Specify Dopaminergic Neurons in *Drosophila*

**DOI:** 10.1371/journal.pone.0026879

**Published:** 2011-11-04

**Authors:** Murni Tio, Joanne Toh, Wanru Fang, Jorge Blanco, Gerald Udolph

**Affiliations:** 1 Neural Development and Repair, Institute of Medical Biology, Singapore, Singapore; 2 Department of Bioengineering, National University of Singapore, Singapore, Singapore; National Institutes of Health (NIH), United States of America

## Abstract

In *Drosophila*, dopaminergic (DA) neurons can be found from mid embryonic stages of development till adulthood. Despite their functional involvement in learning and memory, not much is known about the developmental as well as molecular mechanisms involved in the events of DA neuronal specification, differentiation and maturation. In this report we demonstrate that most larval DA neurons are generated during embryonic development. Furthermore, we show that loss of function (l-o-f) mutations of genes of the apical complex proteins in the asymmetric cell division (ACD) machinery, such as *inscuteable* and *bazooka* result in supernumerary DA neurons, whereas l-o-f mutations of genes of the basal complex proteins such as *numb* result in loss or reduction of DA neurons. In addition, when Notch signaling is reduced or abolished, additional DA neurons are formed and conversely, when Notch signaling is activated, less DA neurons are generated. Our data demonstrate that both ACD and Notch signaling are crucial mechanisms for DA neuronal specification. We propose a model in which ACD results in differential Notch activation in direct siblings and in this context Notch acts as a repressor for DA neuronal specification in the sibling that receives active Notch signaling. Our study provides the first link of ACD and Notch signaling in the specification of a neurotransmitter phenotype in *Drosophila*. Given the high degree of conservation between *Drosophila* and vertebrate systems, this study could be of significance to mechanisms of DA neuronal differentiation not limited to flies.

## Introduction

Asymmetric cell division (ACD) is a fundamental mechanism generating cell fate diversity during nervous system development [1], [2]. In *Drosophila*, progenitor cells delaminate from the neuroectoderm [3] and start dividing along the apical-basal axis in a stem cell-like mode giving rise to another neuroblast (NB) and an intermediate precursor called ganglion mother cell (GMC) [4]. During division, NBs localize proteins such as Inscuteable (Insc) [5] and Bazooka (Baz) [6], [7] to the apical cortex and conversely, proteins such as Numb [8], [9] and Partner of Numb (Pon) [10] to the basal cortex. Pon physically interacts with Numb and directs asymmetric localization of Numb [10]. In general, the apical proteins or protein complexes control the localization of the basal proteins [2]. GMC division is also asymmetric and results in two siblings with distinct cell fates [11].

Early in *Drosophila* embryonic development, the Notch pathway is instrumental in lateral inhibition, a process which singles out NBs from equivalent groups of neuroectodermal cells. During GMC divisions, Notch plays an active role in binary sibling cell fate specification. In this context, two opposing regulators of Notch, i.e. Numb and Sanpodo (Spdo) play important roles. While Numb antagonizes Notch signaling [12] in one daughter cell [13], Spdo promotes Notch signaling in the other sibling [14], [15] resulting in differential activation of Notch signaling which ultimately generates two distinctly specified binary cell fates.

*Drosophila* midline cells arise from a group of mesectodermal cells which separate the mesodermal anlagen from the lateral neurogenic region. Midline cells are characterized by the expression of Single-Minded (Sim), the master regulator of ventral midline development [16], [17]. Midline precursors (MP) express unique or overlapping sets of marker genes [18] and normally divide only once giving rise to two daughter cells.

DA neurons play a fundamental role in health and disease and their loss has been implicated in Parkinson's disease (PD) [19], [20]. In *Drosophila*, DA neurons also have roles in controlling behavior, learning and memory [21]. A hallmark of DA neurons is the expression of Tyrosine Hydroxylase (TH), a rate limiting enzyme in dopamine synthesis [22] and as such TH is commonly used as DA neuronal marker. Although DA neurons in *Drosophila* are known to be present from mid-embryonic development to adulthood, so far mechanistic insights came mostly from studying a single DA neuron derived from the ventral midline [23]. However, the majority of DA neurons in *Drosophila* are of non-midline origin and it has not been demonstrated whether similar mechanisms would apply for DA neuronal specification derived from these neuroblast lineages.

Here, we investigated the developmental origin and molecular mechanisms governing the specification of embryonic and larval DA neurons. We found that ACD and Notch signaling are crucial mechanisms for specifying DA neurons. In this context, Notch signaling represses DA neuronal fate or in other words DA neurons differentiate from cells without active Notch signaling. Our study provides for the first time a link between ACD, Notch signaling and DA neuron specification in *Drosophila*. Studying the cellular and molecular mechanisms of DA neuron specification in *Drosophila* might provide useful insights into vertebrate systems which could ultimately support strategies for controlling *in vitro* cell fate specification of DA neurons.

## Materials and Methods

### *Drosophila* strains

*Drosophila* strains used were *y w^1118^* (used as *wild type*), *Notch^55e11^*, *Notch^ts1^*, *spdo^G104^* [14], *numb^1^* [13], *insc^22^* [5]. Lac-Z lines used were *P(PZ)wg^02657^ cn^1^/Cyo ; ry^506^* (Bloomington Stock Center) and *hkb-lacZ^5953^/ hkb-lacZ^5953^*. *GAL4* and *UAS* lines used were *ple-GAL4* [24], *sim-GAL4* [25] and *da-GAL4* (Bloomington Stock Center), *UAS-numb* [26], *UAS-Notch-intra* [27],*UAS-GFP* (Bloomington Stock Center) and *UAS-bazRNAi* (VDRC). Fly strains used for generating MARCM clones were (1) MARCM ready strains: *y, w*, *hs-FLP/y, w*, *hs-FLP* ; *P[neoFRT]40A, tubP-GAL80/P[neoFRT]40A, tubP-GAL80 ; tubP-GAL4, UAS-mCD8::*GFP*/TM6B, Hu, Tb* and *y, w*, *hs-FLP/y, w*, *hs-FLP* ; *P[neoFRT]42D, tubP-GAL80/ Cyo, Act-GFP; tubP-GAL4, UAS-mCD8::*GFP*/TM6B, Hu, Tb* and *y, w*, *hs-FLP/y, w*, *hs-FLP; tubP-GAL4, UAS-mCD8::GFP/Cyo, Act-GFP ; P[neoFRT]82B , tubP-GAL80 / TM6B, Hu, Tb* (2) Strains with FRT sites used to recombine the mutant chromosomes and as controls were: *w[1118]; P36F P{ry[+t7.2] = neoFRT}40A*, *P{ry[+t7.2] = neoFRT}42D; ry[605]*and *P[neoFRT]82B e^s^ spdo^G104^/ TM3 Sb^1^* (Bloomington Stock Center). The *bazooka UAS-siRNA* line used was *w^1118^; P[GD1384]v2915* (VDRC).

### Lineage analysis

The lineages and projection patterns of TH-positive neurons were traced using the flip-out technique [28], [29]. Briefly, embryos were collected for 3 hours from a cross between flies of genotypes P{UAS-mCD8::GFP.L}LL4, P{hsFLP}22, y^1^ w^*^; Pin^1^/CyO and *w^1118^; P{AyGAL4}25/CyO*. This was followed by three hours of aging and after which a 10-minute exposure to heat shock (32°C) to generate GFP positive clones in flies carrying both the flippase and FRT components. For lineage analysis in the embryos, heat treated embryos were further aged at 18°C until end of embryogenesis before fixation while for lineage analysis in the larva brain, heat treated embryos were aged at 18°C until third larval instar stage followed by fixation. Clones which were positive for both GFP and TH immunoreactivity were then analyzed and compared to published lineage data [30], [31], [32].

### MARCM and siRNA knock-down analysis

For the clonal analysis, *numb* and *insc* mutations were recombined into the respective FRT chromosomes. Individual cross was set up between the MARCM ready strain and the strain carrying a mutation and an FRT site on same chromosome. Clones were induced between 4 to 7 hours (at 25°C) of embryonic development by heat shocking the embryos for 30 minutes at 37°C as described [33], [34]. Heat treated embryos were further aged at 25°C until third larval instar stage when larval brains were dissected. Clusters containing GFP marked clones were analyzed for TH expression. For the control experiments, crosses were set up between the MARCM ready strains and the strains containing FRT sites followed by analysis of TH expression. For siRNA knock-down analysis, *UAS-siRNA^baz^* was expressed using a ubiquitous driver, *Da-GAL4*. Third larval instar brains were dissected and analyzed for TH expression.

### Conditional *Notch* knock-down experiments

For the analysis of *Notch* functional requirement in the embryonic H-cells, *Notch* embryos were collected for 1 hour at permissive temperature (18°C), aged for 5/7/9 hours at 18°C and exposed to restrictive temperature (29°C–30°C) in a water bath for 2 hours. Samples were further aged at 18°C until embryonic stages 16–17 before fixation. In a separate experiment, five-hour aged embryos were exposed to restrictive temperature continuously until embryonic stages 16–17 and analyzed. Dissection of period of Notch requirement for neuronal fate specification of the larval DA neurons was carried out by segregating out embryos of different developmental stages as well as early first larval instar and exposed them separately to restrictive temperature for 2 hours, shifted back to permissive temperature until third larval instar stage when the larval brains were dissected and analyzed. For the controls, embryos were collected from *N^ts^* flies which were constantly grown at 18°C.

### Immunohistochemistry

Embryo fixation and immunostaining were done as previously described [35]. Larval brains were fixed with 4% formaldehyde for 30 minutes and stained similarly as in embryos. Primary antibodies used were: rabbit α-GFP (1∶100, Clontech), mouse α-β-galactosidase (1∶ 3,000, Sigma-Aldrich), rabbit α-β-galactosidase (1∶ 2,000, MP Biomedicals), mouse α-mTH (1∶100, Chemicon), rabbit α-dTH (1∶250, [36]), rabbit α-Inscuteable (1∶500, [35]), rabbit α-Pon (1∶500, [10], rabbit α-Period (1∶1,000, [37]), mouse α-Neurotactin/BP106 (1∶10, DSHB). FITC and Cy3 fluorescence conjugated secondary antibodies used were from Jackson ImmunoResearch. After staining, images were taken using Olympus confocal microscopes.

## Results

### Dopaminergic neurons in the embryonic and larval CNS

**Figure 1:**
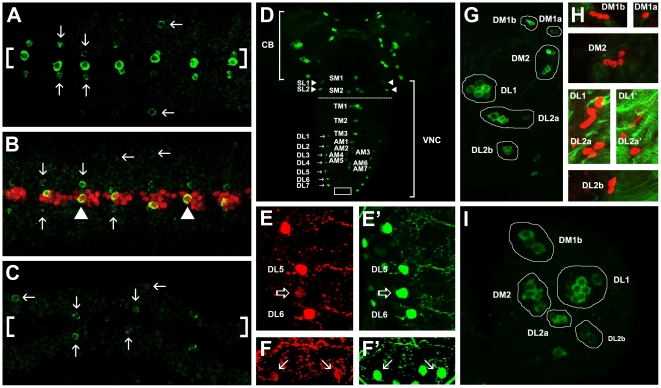
Development of DA neurons. (**A–C**) Ventral views of St16–17 embryos. Anterior is to left. (A) Embryo showing TH expression in H-cells at the ventral midline (bracketed). TH also transiently labels two paramedial cells (vertical arrows) as well as one cell per hemineuromere at dorsal lateral positions (horizontal arrows). (B) Co-localization of TH (green) and β-Galactosidase under regulation of *sim-GAL4* (red) showing midline origin of H-cells (arrowheads). Note that the paramedial and dorsal lateral DA neurons do not express Sim-LacZ and hence are not of midline origin. (C) In *sim* mutants, TH expression is completely abolished in H-cells but unaffected in paramedial and dorsal lateral cells. (D) Third larval instar brain showing DA neurons in the central brain (CB) and ventral nerve cord (VNC). 12 H-cells are located at the midline of the VNC from anterior to posterior direction, two cells (SM1 and SM2) in the suboesophageal, three cells (TM1–TM3) in the thoracic and seven cells (AM1–AM7) in the abdominal neuromeres. The two cells neighboring SM2 and TM1 are termed SML2 and TML1, respectively. At each lateral position, two SL neurons (SL1 and SL2, arrowheads) are located at the suboesophageal neuromeres and seven TH-positive DL neurons span the thoracic and abdominal segments. (E–F) TH and (E′, F′) GFP expression under regulation of *ple-GAL4*. Note that between DL5 and DL6, one weakly TH expressing cell is detected which expresses GFP (open arrows in E and E′). Near the posterior-most tip of the ventral ganglia (boxed region in D), a pair of weakly TH expressing cells can be detected which also express GFP (arrows in F and F′). (G) Magnified view of left lobe of CB in (D). Six major DA neuronal clusters are observed: DM1a (1 cell), DM1b (3 cells), DM2 (4 cells), DL1 (7 cells), DL2a (4 cells) and DL2b (2 cells). (H) The DA clusters only express TH (red) but not Neurotactin (green). (I) Central brain of early first larval instar showing five of six DA neuronal clusters: DM1b (3 cells), DM2 (4 cells), DL1 (7 cells), DL2a (3 cells) and DL2b (2 cells). Note that DM1a and one neuron in DL2a cluster do not express TH at this stage. SM: suboesophageal medial, TM: thoracic medial, AM: abdominal medial, SL: suboesophageal lateral, SML: suboesophageal mediolateral, TML: thoracic mediolateral, DM: dorsal medial and DL: dorsal lateral.

We began our analysis by examining the developmental profile of major clusters of DA neurons from embryonic until third larval instar stages. Tyrosine Hydroxylase (TH), encoded by the *Drosophila pale (ple)* gene [38] is a rate limiting enzyme for the synthesis of neurotransmitter dopamine [22] and therefore is widely used as a phenotypic marker for DA neurons. Using α-TH antibody as well as *ple-GAL4 ; UAS-GFP* reporter system [24] we analyzed TH expression from embryonic to larval stages of development. In the embryo, TH protein expression was first observed at stage 14 (St14) in one neuron per segment in the ventral midline representing the H-cell [23]. At St15, additional TH expression was observed weakly in a single neuron per hemineuromere at the dorsal lateral positions as well as in two paramedial cells neighboring the H-cells. By St17, TH expression became much more prominent in cells of the VNC; in 12 midline H-cells spanning the anterior-most suboesophageal segment-1 to the posterior-most abdominal-8 (A8) segment, in one cell per hemineuromere at dorsal lateral positions and transiently in two small paramedial (PM) cells flanking the H-cells (Figure 1A, see also [39]). The two PM cells at suboesophageal segment-2 and thoracic segment-1 (T1) were slightly bigger in size than the other PM cells at T2 to A8. In agreement with a previous report [39], we observed TH expression in the embryonic brain lobes at St17 although expression was weak and in only a subset of cells normally labeled in late larval stages.

The H-cell is derived from the MP3 midline progenitor which produces the dopaminergic H-cell and its glutaminergic sibling, H-cell sib [23]. Midline origin of H-cell was confirmed by the expression of β-Galactosidase under the regulation of Sim-GAL4, a midline specific driver (Fig. 1B) and the loss of H-cells in *sim* mutants (Fig. 1C). Neither the paramedial nor the dorsal lateral DA neurons were of midline origin as they did not co-express Sim-lacZ and were unaffected in *sim* mutants (Fig. 1B, C).

Embryonic TH expression persisted into larval stages. In the midline of third larval instar VNCs, H-cells could be found at the suboesophageal segments (SM1 and SM2), thoracic segments (TM1 to TM3) and abdominal segments (AM1 to AM7) (Fig. 1D; nomenclature according to Selcho et al. [40]). The two cells directly neighboring SM2 and TM1 were referred to as suboesophageal mediolateral (SML2) and thoracic mediolateral (TML1) DA neurons. Nine strongly TH expressing cells could be found on each side at the lateral positions: two at the suboesophageal regions (SL1 and SL2) (Fig. 1D) and seven at the thoracic and abdominal regions (DL1–DL7) (Fig. 1D). We also observed an additional TH expressing cell at each dorsal lateral position between DL5 and DL6 (Fig. 1E, E′) as well as two TH-positive cells at the posterior-most tip of the VNC (Fig. 1 F, F′), however they were not analyzed further due to relatively weak TH expression levels.

In the central brain of third larval instar, four major groups of DA neurons were previously reported spanning dorsal medial (DM) and dorsal lateral (DL) positions of each brain hemispheres [39], [41]. Recently, a report described the projection patterns of individual neurons within each clusters in greater detail and it seemed that although individual neurons within each clusters shared fasciculation pattern, they might not share similar projection patterns [40]. For simplicity and ease of further analysis, we regrouped the DA neurons into a cluster based on level of proximity to one another and similarity in axonal fasciculation patterns (also as an indication for a possible lineage relationship) (Fig. S1). Using this approach, we regrouped the DA neurons into 6 clusters per hemispheres. Three clusters of neurons were clearly separated from one another at dorsal medial positions and neurons in these clusters generally projected ipsilaterally: DM1a, which contained a single neuron; DM1b which contained three neurons and DM2 which contained four neurons. The three distinct clusters in the dorsal lateral positions were: DL1 which contained seven tightly grouped neurons having axonal projection across the midline to the contra-lateral side of the brain hemisphere; DL2a which consisted of four neurons, some of which arborized on both sides of the brain but some remained strictly at the ipsilateral side of the brain. The DL2b cluster consisted of two neurons projecting towards the midline and after crossing the midline terminated on the contra-lateral side of the brain where they also showed some ipsilateral and descending projections to the suboesophageal neuromeres (Fig. 1G and Fig. S1; see also Selcho et al. [40]).

All larval DA neurons did not express Neurotactin (Fig. 1H), a marker that specifically labels secondary neurons born only during larval neurogenesis [42]. This clearly demonstrated that DA neurons in the larval hemispheres were born during embryonic development. Although it was reported that DA neurons in the larval hemispheres already expressed TH at the end of embryogenesis (St17) [39], individual clusters of DA neurons were not studied in greater detail. At early first larval instar (L1) the composition of all the DA neuronal clusters was almost complete with the exceptions of the DA neuron in DM1a as well as one neuron in the DL2a cluster which still did not express TH at L1 (Fig. 1I). By second larval instar however, the compositions of all six DA neuronal clusters were indistinguishable from those seen in third larval instar.

In summary, DA neurons in *Drosophila* were present at the embryonic VNC in segmental patterns as one cell per neuromere in the midline and generally one cell per hemineuromere in the paramedial as well as dorsal lateral positions. In the larval brain, a total of 76 dopaminergic neurons which strongly expressed TH and were mostly of embryonic origin could be found. The VNC contained a total of 34 DA neurons of which 12 were H-cells of midline origin and spanned the VNC from anterior to posterior. In the suboesophageal region, there were two neurons at the mediolateral positions (SML2) and four DA neurons at the lateral positions (SLs). Two neurons could be found at mediolateral positions in the first thoracic segment (TML1) and fourteen DA neurons at the dorsal lateral positions (DLs) in the abdominal segments. Both larval hemispheres contained a total of 42 DA neurons (2×21 cells) organized in six distinct clusters based on their physical proximity and axonal fasciculation patterns.

### Analysis of NB lineage context of DA neurons in the VNC

**Figure 2:**
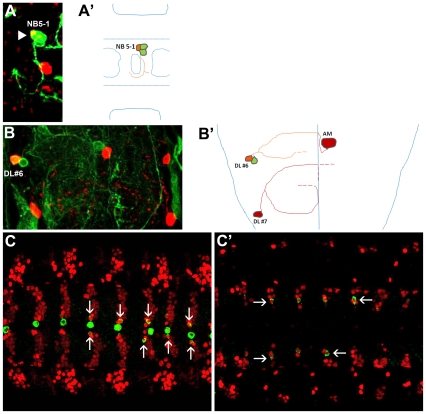
Lineage analysis of paramedial and dorsal lateral DA neurons. (A, B) are images of GFP-labeled clones (green) which contain TH-positive DA neurons (red). (A′, B′) are the corresponding schematic representations of NB clones shown in A and B. (A) A 3-cell GFP-labeled abdominal clone which contains a paramedial cell that expresses TH (arrowhead). Neurons from this clone fasciculate together and project towards the midline but bifurcate at the contralateral connective. Such clone composition and the projection patterns are typical for neurons that arise from NB5-1 lineage (A′). (B) A 2-cell GFP-labeled clone consisting of a TH-positive dorsal lateral DA neuron (orange) containing axons that bifurcate and project towards the midline (B′). Axonal projections which could not be followed completely are marked by dashed lines. (C, C′) Ventral and dorsal projections of confocal sections showing paramedial (vertical arrows in C) and dorsal lateral (horizontal arrows in C′) DA neurons, expressing both Wingless (red) and TH (green).

Except for the H-cells, information regarding the lineage context of other embryonic DA neurons in the VNC is unavailable. Hence, we performed flippase-induced mitotic recombination to generate GFP-labeled NB clones which were co-labeled with TH to determine the NB lineages giving rise to the VNC DA neurons. By comparing such flippase induced NB clones with published lineage data [30], [31], [32], we found four clones consisting of an average of 3 to 4 inter-neurons located ventrally to the neuropile which also contained the paramedial DA neurons (Fig. 2A). Two such clones were at the abdominal segment-1 (A1, data not shown) consisting of 3 GFP marked interneurons which fasciculated and projected as a single bundle towards the ventral midline and extended their neurites contra-laterally across the posterior commissures. Two examples of clones in A4 (Fig. 2A, A′) also consisted of 3 GFP marked interneurons which fasciculated together but bifurcated at the contra-lateral connectives. From its small lineage, position within the ventral nerve cord as well as unique projection patterns of neurons, we thus conclude that the paramedial DA neurons are progeny of NB5-1 [32].

**Figure 3:**
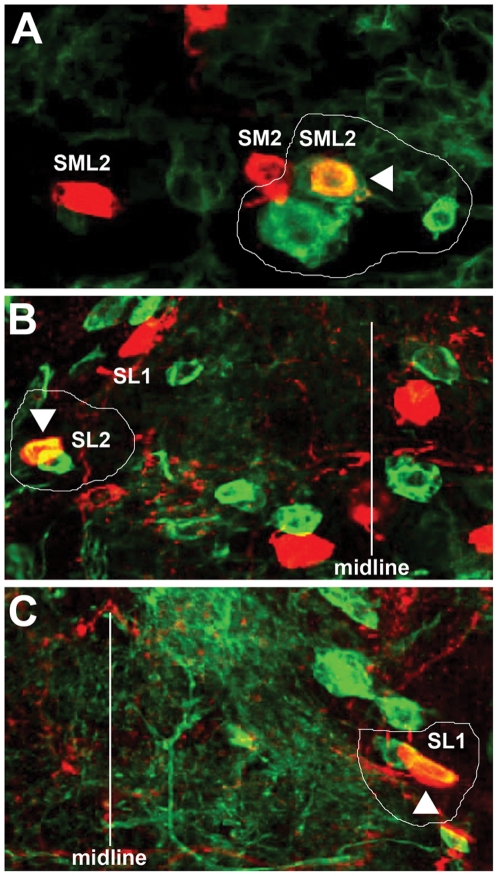
SML2 as well as SL1 and SL2 DA neurons are derived from individual NBs with small lineages. (A, B and C) are images of GFP-labeled clones (green) which contain TH-positive DA neurons (red) in the SML2 (A), SL2 (B) and SL1 (C) clusters. (A) SML2 neuron is born from a NB that gives rise to about 4 cells. (B) SL2 DA neuron is born from a NB that gives rise to 2 cells. (C) SL1 DA neuron is born from a NB that gives rise to 2 cells. In all panels, arrowheads mark the cells that show colocalization of both TH and GFP. All clones are encircled and vertical lines (B, C) mark the approximate positions of the midline.

We also obtained abdominal clones which contained the dorsal lateral DA neurons. However, due to late appearance of TH expression in these cells, we could not assign them unambiguously to a particular NB. Those clones consisted of 2 to 3 GFP marked neurons with axons projecting towards the midline but bifurcated before reaching the midline. Also, a subperineural glia (SPG) was part of the clone. Such features are typical of the NB5-6A lineage [31]. Analysis of clones in the larva further suggested that the dorsal lateral DA neurons arose from small lineages of 2–3 cells (Fig. 2B, B′; see also Table 1). To further confirm lineage identity of the paramedial and dorsal lateral DA neurons to row 5 NBs, we analyzed whether DA neurons co-expressed Wingless-LacZ (Wg-LacZ) or Huckebein-LacZ (Hkb-LacZ) as Wg-LacZ is known to be expressed in row 5 NB and within row 5, Hkb-LacZ expression is limited to NBs 5-4 and 5-5 NBs [43], [44]. We found that both the paramedial and the dorsal lateral DA neurons expressed Wg (Fig. 2C, C′) but not Hkb (data not shown). Expression of Wg-LacZ by the dorsal lateral DA neurons suggested that these neurons were derived from row 5 NBs and of these NB5-1, NB5-2, NB5-3 and NB5-6 do not express Hkb-LacZ. As NB5-1, NB5-2 and NB5-3 are located close to the ventral midline while the lateral DA neurons are located most laterally, they are thus possibly derived from NB5-6. For technical reasons, lineage analysis of DA neurons in the suboesophageal and first thoracic neuromeres was carried out in the larva. We found that SML2 (Fig. 3A) and SL neurons (Fig. 3B, C) were derived from small NB lineages which only contained 3 to 4 neurons and 2 to 3 neurons, respectively (see Table 1). However, due to the increased complexity of axonal projection patterns of larval VNC neurons, we were unable to assign these groups of DA neurons to any known and described NB lineages. Thus, the DA neurons in the fly embryonic ventral nerve cord are derivatives of the midline progenitor MP3, NB5-1, possibly the abdominal variant of NB5-6 (NB5-6A) and two other yet to be identified small NB lineages.

**Table 1:** Lineage context of DA neurons in the larval VNC.

*Clusters*	*n*	*No. GFP+ cells/clone*	*No. TH+ cells*
DL	24	1.8±0.2	1
SL1	3	2±0	1
SL2	3	2.7±0.3	1
SML2	5	3.6±0.4	1
TML1	1	4	1

DL, SL1, SL2, SML2, and TML1 DA neurons derive from relatively small NB lineages. DL, dorsal lateral; SL, suboesophageal lateral; SML, suboesophageal mediolateral; TML, thoracic mediolateral.

### Role of asymmetric cell division in the specification of DA neurons

**Figure 4:**
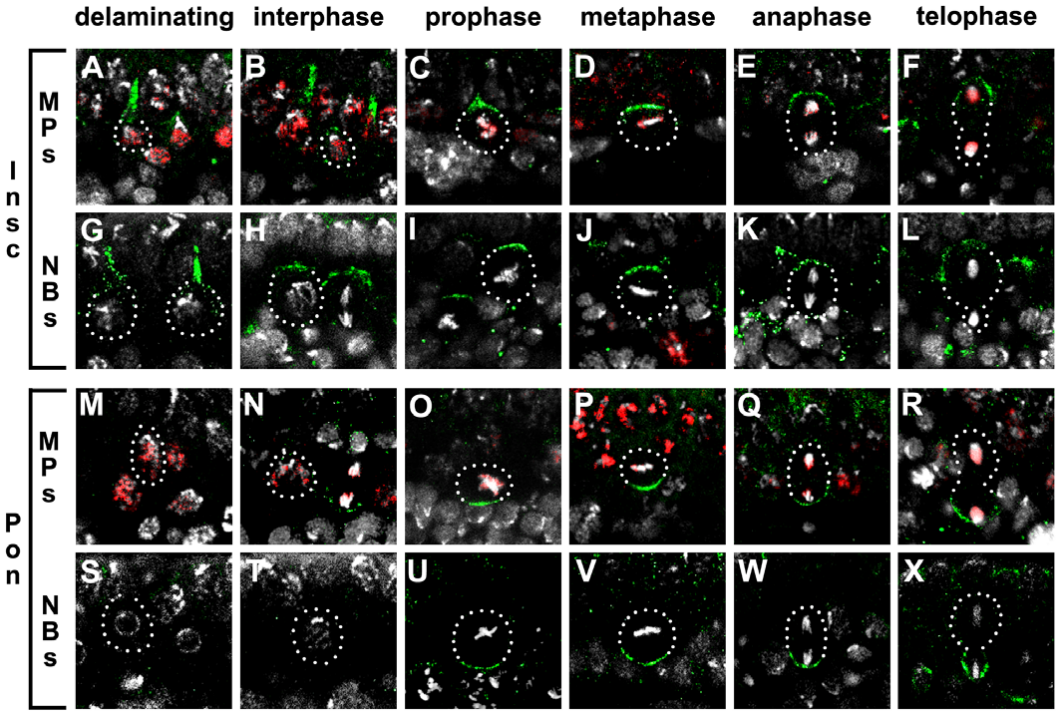
Asymmetric protein localization in MPs and NBs. (A–L) Insc localization in MPs (A–F) and NBs (G–L). (M–X) Pon localization in MPs (M–R) and NBs (S–X). Note that at all phases of mitosis, Insc and Pon are asymmetrically localized in most MPs in similar fashion to that of NBs as well as having the plane of division oriented along the apical basal axis. In all panels, Insc and Pon are shown in green and MPs are marked by the expression of Sim-LacZ (red). Topro-3 (white) stains DNA. Apical is up and basal is down. MP: midline progenitor; NB: neuroblast.

A role of a basally localized asymmetric component such as Numb in fate specification of daughter cells derived from the midline precursor cells (MPs) was recently reported [23], [45]. To extend on these findings, we first analyzed asymmetric localization of an apical complex protein Inscuteable (Insc) and a basal complex protein Partner-of-Numb (Pon) in MPs of St10–11 embryos. We found that Insc was generally expressed at the apical cortex of MPs throughout MP divisions (Fig. 4A–F). Reversely, Pon was observed at the basal cortex of MPs from prophase to telophase when it was specifically distributed to the basal daughter cells following cytokinesis (Fig. 4M–R). The localization patterns of Insc and Pon in MPs as well as their apical-basal polarity were similar to those seen in the NBs (Fig. 4G–L and Fig. 4S–X, respectively), suggesting that MPs divide asymmetrically in a similar manner as described for NBs. There were a few exceptions in which NBs did not express Insc though they divided perpendicular to the cell surface. As Insc restricts proteins such as Numb to the basal cortex of dividing NBs or GMCs, its absence may result in distribution of Numb to both daughter cells hence resulting in equalized daughter cell fates. Symmetric division has been reported for the MP1 [23]. In addition, we also observed MPs which divided parallel to the cell surface and hence would likely distribute Insc to both daughter cells.

**Figure 5:**
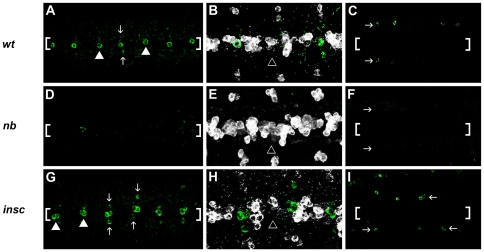
Insc and Numb affect DA neuronal specification. (A–I) St16–17 embryos showing TH expression in green (all panels) and Per expression in white (B, E, and H). (B, E and H) represent magnified and merged frames of A, D, and G, respectively. (A–C) *wt* embryos. One H-cell can be seen (A, arrowheads) flanked by two paramedial cells (vertical arrows). Per expression is present in cells at the midline as well as outside of the midline (B, in white) but excluded from the H-cells. TH expression in the dorsal lateral cells near the lateral-most border of the CNS (C, horizontal arrows). In *numb* embryos (D–F), H-cells at the ventral midline (D, bracketed) as well as dorsal lateral cells (F, horizontal arrows) are mostly missing. The loss/reduction of H-cells is accompanied by the gain of Per expression (E, open arrowhead). The paramedial cells are generally undetectable in *numb* embryos. In *insc* embryos (G–I), H-cells are duplicated at high frequency (G, arrowheads) but the paramedial cells are generally unaffected (G, vertical arrows). The gain of H-cells is accompanied by the reduction of Per expression (H, open arrowhead). The dorsal lateral DA neurons are duplicated but at lower frequency than that of H-cells (I, horizontal arrows). Brackets (A, C, D, F, G and I) mark the approximate positions of the midline.

To investigate whether DA neurons were affected when the asymmetric cell division machinery was disturbed, we analyzed TH expression in *numb* and *inscuteable (insc)* mutant embryos. In wild type, a single H-cell was found in each neuromere at the ventral midline (100%±0, n = 96 neuromeres, Fig. 5A). While in *numb* mutants H-cells were not observed in most neuromeres (78%±3.7, n = 125; Fig. 5D), in *insc* mutants they were duplicated at high frequency (73.6%±3.5, n = 186 neuromeres; Fig. 5G). To further support a sibling cell fate transformation, we analyzed the expression pattern of Period (Per) which is expressed by the H-cell sibs, the iVUMs as well as other non-midline derived cells (Fig. 5B) [18]. We found that the loss and gain of H-cells in *numb* and *insc* mutant embryos were accompanied by the reverse changes in the number of Per expressing cells at medial positions (Fig. 5E and H, respectively). These data suggested a fate transformation between H-cell and its sibling involving asymmetric cell division. Cell fate transformation in *numb* and *insc* mutations were also observed for the other VNC DA neurons and their siblings such as the dorsal lateral DA neurons, which were mostly lost in *numb* (Fig. 5F) and duplicated in *insc* although duplication was at a low frequency (14.2%±3, n = 201 hemineuromeres; Fig. 5I). We also analyzed the paramedial DA neurons and found that they were generally unaffected in *insc* mutants. By analyzing clear TH-expressing paramedial DA neurons in late staged embryos which also concurrently showed duplicated H-cells, we observed 1 TH-positive cell in most hemineuromeres (99.2%±0.6, n = 240, Fig. 5G). However, these cells were generally absent in *numb* mutants (Fig. 5D).

**Figure 6:**
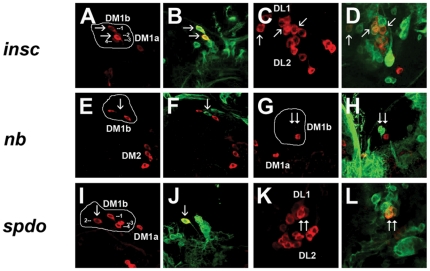
*insc^−^, numb^−^ and spdo^−^* affect specification of DA neurons in larval brain hemispheres. (A–D) *insc*, (E–H) *numb* and (I–L) *spdo* clones were generated using the MARCM system. A, C, E, G, I, and K are Z-stacks showing TH expression (red) and B, D, J and L represent selected merged frames showing clear colocalization of TH (red) and GFP (green) in a few cells of the mutant clones. Note the increased numbers of cells at DM1b (numbered 1 to 4) and DL1 clusters in *insc^−^* clones (A–D). Reversely, in *numb^−^* clones the cluster sizes are reduced as shown for the DM1b clusters (E–H). Arrows in (E) and (G) point to loss of TH expression at the same positions showing GFP expression (F and H). In *spdo^−^* clones, increased number of TH expressing cells are seen for DM1b (I, J, numbered 1 to 4) and DL1 (K, L) clusters. Mutant clones are marked by GFP expression. For ease of identification, the affected DM1b clusters in all three mutants are encircled. In all panels, other nearby wild type TH clusters may not be in the same focal plane.

**Figure 7:**
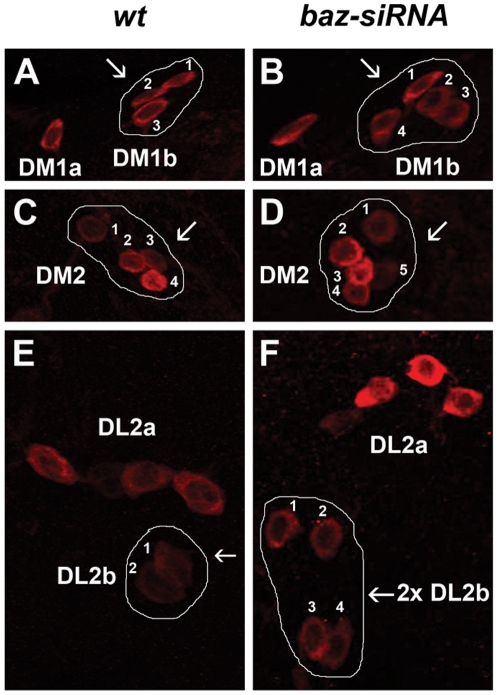
*bazooka* knockdown results in additional DA neurons in the larval brain hemispheres. Third instar larval brains of genotype *UAS-siRNA^baz^/+ ; da-GAL4/da-GAL4* are shown. (A, C and E) represent *wt*, (B, D and F) shows *baz* knockdown. An increase of 1 cell is observed in DM1b (B) and DM2 (D) clusters. However, duplication of the entire DL2b cluster occurs frequently (F) in *baz* knockdown. Wild type and affected mutant clusters are encircled and cell numberings are included.

More than half of larval DA neurons are found in the central brain hemispheres. Therefore, we proceeded to study the involvement of ACD in fate specification of these neurons. As most mutations affecting asymmetric cell division (ACD) are embryonic lethal, we took two alternative approaches: firstly, we generated MARCM labeled mutant clones [33], [34] and secondly, we knocked-down gene function using the siRNA approach [46], [47]. MARCM clones were generated for *insc* and *numb* mutations as well as their respective FRT strains which served as controls and the DA clusters containing GFP-labeled clones were analyzed for the total number of TH expressing cells (see Table 2). We found that generally the numbers of DA neurons increased when DA clusters were part of *insc* MARCM clones. For example, we found that DM1b contained 4 TH-positive cells (n = 2) (Fig. 6A, B), DL1 which contained an average of 11.5 TH-positive cells (n = 4) (Fig. 6C, D) and DL2a which contained 5 TH-positive cells (n = 1) (Table 2). We were unable to obtain *insc* clones that overlapped with DM1a, DM2 and DL2b clusters. Reversely, when *numb* clones were found in the vicinity or overlapped partly with DA clusters, less DA neurons were observed; e.g. the DM1a cells were absent (n = 2), DM1b which comprised of an average of 1.5 TH-positive cells (n = 4) (Fig. 6E–H), DL2a which comprised of an average of 2.7 TH-positive cells (n = 3) and DL2b which comprised of 1 TH-positive cell (n = 1) (Table 2). It needs to be noted that due to technical limitations it was not possible to unambiguously link the GFP-positive mutant cells to missing TH+ neurons. However, our conclusion that *numb* affects larval DA neuron specification is based on the observation that the number of DA neuron was reduced in the DM1b clusters when the GFP-positive mutant cells were part of or in close proximity with the other wild type TH+ cells in these clusters. In addition, the mutant GFP+ cells occupied a position where the missing DA neurons were expected to be located, suggesting that the GFP-positive cells could represent the missing TH-positive cells. A role for *numb* in larval DA neuron specification was also supported by the finding that in embryos of *numb* mutants DA neurons were largely missing (see above). We did not obtain *numb* clones that overlapped with DM2 and DL1 clusters. We also examined *sanpodo* (*spdo*) clones and found an increase of DA neurons with the exception of the DL2a cluster. For example, DM1a comprised of 2 TH-positive cells (n = 1), DM1b which comprised of 4 TH-positive cells (n = 3) (Fig. 6I, J), DM2 cluster which contained 5 TH-positive cells (n = 1), DL1 cluster which contained 11 TH-positive cells (n = 1) (Fig. 6K, L) and DL2b which comprised of an average of 3.7 TH-positive cells (n = 3) (Table 2). In the VNC, the SML2 (Fig. S2B, B′) and TML1 (Fig, S2C, C′) cells were duplicated in *spdo* mutant clones (n = 4). Ubiquitous functional knock-down of *bazooka (baz)* using *da-GAL4* also resulted in an increase of TH-expressing cells in DM1b (Fig. 7B), DM2 (Fig. 7D) and DL2b clusters (Fig. 7F) as well as the lateral cells in the suboesophageal regions (SLs) of the VNC (data not shown). Together, the MARCM analysis as well as siRNA knock down experiments clearly demonstrated a role for ACD in fate specification of DA neurons in *Drosophila*.

**Table 2:** Quantification of MARCM clones for *insc*, *nb* and *spdo* mutations.

			*Clusters*			
*Genotypes*	DM1a (1)	DM1b (3)	DM2 (4)	DL1 (7)	DL2a (4)	DL2b (2)
*insc*	n.d.	4 cells (n = 2)	n.d.	8, 9, 13, 16 cells (n = 4)	5 cells (n = 1)	n.d.
*nb*	0 cells (n = 2)	1, 1, 2, 2 cells (n = 4)	n.d.	n.d.	2, 3, 3 cells (n = 3)	1 cell (n = 1)
*spdo*	2 cells (n = 1)	4 cells (n = 3)	5 cells (n = 1)	11 cells (n = 1)	n.d.	3, 4, 4 cells (n = 3)

Removal of genes involved in asymmetric cell division alters number of DA neurons in clusters. The wild type numbers of cells in clusters are in brackets. n, number of cases observed; nd, not determined.

### Notch suppresses DA neuronal specification

Notch has been described as an effector of asymmetric cell division and binary sibling cell fate resolution [11], [48], [49]. To investigate the role of Notch signaling in the specification of embryonic DA neurons, we first analyzed TH expression in the ventral midline of *Notch^55e11^* mutants and found supernumerary TH expressing cells. On average, we detected 6.6±0.2 H-cells in each segment (n = 93; Fig. S3B). The dorsal lateral DA neurons were also affected and on average 2.9±0.2 TH-positive cells were found per hemineuromeres (n = 132, Fig. S3B). In the embryonic ventral midline, the Period protein is expressed in H-sibs and iVUMs [18] (Fig. S3C). Hence, we investigated if the increase of H-cells was accompanied by a reduction of Period expressing H-sibs. We found that loss of *Notch* resulted in complete loss of Period expression in all cells (Fig. S3D).

**Figure 8:**
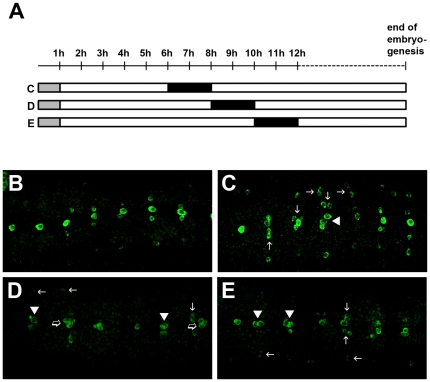
Loss of Notch functions results in additional DA neurons. (A) Graphical representation of the experimental paradigm. Grey boxes mark the period of egg collection at 18°C, white boxes mark the period of rearing at 18°C and black boxes mark the period of exposure to non-permissive temperature (29°C–30°C), hence removal of Notch function. C, D and E on the left side pair with the corresponding panels. (B–E) Ventral views of St16–17 embryos. (B) *wt* and (C–E) *N^ts^* embryos exposed to non-permissive temperature for 2 h after 5–6 h (≈St8, C), 7–8 h (St9–10, D) and 9–10 h (≈St10–11, E) of development at 18°C. (C) Removal of Notch function at around St8 results in duplication of H-cells in the midline (arrowheads), paramedial cells (vertical arrows) and dorsal lateral DA neurons (horizontal arrows). (D) Removal of Notch function at St9–10 results in duplication (arrowheads) and some triplication (open arrows) of the H-cells. (E) Removal of Notch function at St10–11 mostly results in duplication of H-cells (arrowheads). Note that Notch functional removal at the later time points (St9–11) does not affect specification of the dorsal lateral DA neurons (horizontal arrows in D and E) although duplication and triplication of the paramedial cells are still observed at high frequency (vertical arrows in D, E). h, development stage in hours.

Previously, it was reported that disruption of the Notch signaling pathway caused transformation of some MPs towards MP3 fate which then resulted in supernumerary H-cells [23]. Although the phenotype indicated a role of Notch on MP3 fate specification, a role of Notch on binary cell fate specifications has not been clearly demonstrated and not much is known about the temporal requirement for Notch in MP3 fate and sibling cell fate specifications. Therefore, we dissected the two roles of Notch with a conditional knock-out approach using the *Notch* temperature sensitive allele (*N^ts1^*). A typical midline progenitor with the exception of the midline neuroblast (MNB) divides only once at St8 when the eight midline progenitors per segment generate about 16 midline cells including the H-cell and the H-cell sib neurons [23], [50]. Generally, embryos were collected for 1 h and further grown on 18°C for specific periods (5 h, 7 h or 9 h), exposed to non-permissive temperature for 2 h to remove Notch function at particular stages and were then reared until St17 with Notch function restored (Fig. 8A). When *Notch* function was removed for 2 h after 5 to 6 h of embryonic development at permissive temperature (≈St8) which was developmentally much earlier than the division of MPs during St10–11when binary post-mitotic siblings were generated, 12.4% of neuromeres (n = 97) showed duplicated H-cells and 1% of segments contained more than 2 H-cells (Fig. 8C; Table 3). This suggested that removal of *Notch* at this developmental period possibly affected the process of MP specification and loss of Notch resulted in additional MP3s. The dorsal lateral DA neurons were also duplicated (Fig. 8C) in about 23% of the hemineuromeres (n = 165) whereas the paramedial cells were found to be either duplicated (15%) or triplicated (2%) (n = 138 hemineuromeres) (Fig. 8C). When *Notch* function was removed for two hours after 7 to 8 hours of embryonic development (≈St9 to early St10), 1 H-cell (26%), 2 H-cells (57%), 3 H-cells (12%) and 4 H-cells (4%) (n = 298 neuromeres) were observed (Fig. 8D; Table 3). The high frequency of H-cells duplication suggested that binary cell fate specification involving Notch signaling in the MP3 lineage was disturbed although the presence of more than two H-cells suggested that at this same time point MP3 specification was also affected. Removal of *Notch* at these stages also resulted in additional paramedial (PM) cells: 1 PM (19%), 2 PMs (50%), 3 PMs (29%) and 4 PMs (2%) (n = 238 hemineuromeres). When *Notch* function was removed for 2 hrs after 9 to 10 hours of embryonic development (≈early St10 to St11), 1 H-cell (18%), 2 H-cells (71%), 3 H-cells (6%) and 4 H-cells (5%) were observed (Fig. 8E and Table 3). The high frequency of duplicated H-cells in conjunction with the lower number of neuromeres having more than two H-cells suggested that at this developmental phase Notch was predominantly affecting binary sibling cell fate specification. The paramedial cells were also affected with 1 PM (28%), 2 PMs (47%), 3 PMs (20%) and 4 PMs (4%). However, removal of Notch at St9–11 of embryonic development did not seem to affect the dorsal lateral DA neurons (Fig. 8D, E and Table 3), suggesting that at this developmental phase Notch was not required for the specification of these neurons. Thus, our data suggested temporal requirement for Notch on MP3 specification and/or maintenance of MP3 fate suppression at around St8 to early St10 and a requirement for Notch in binary sibling cell fate specification at St9–11. Conversely, when we over-expressed the constitutively active intracellular domain of Notch (N^intra^) in midline cells using *sim-Gal4*, we found a complete loss of DA neurons (Fig. S4B, E). Similar to a previous report by Wheeler et al. [23], over-expression of Numb with *sim-Gal4* resulted in duplication of H-cells (Fig. S4C, F). SML2 and TML1 (arrows in Fig. S4B, C) were not affected in these experiments, suggesting that these cells were not of midline origin. As the additional TH expressing cells in the midline phenocopied the projection pattern of the H-cell (data not shown), complete transformation of H-sib into H-cell was likely. Loss of H-cells in the embryo following over-activation of Notch signaling was also accompanied by loss of the corresponding axonal projections.

**Table 3:** Temporal analysis of *Notch* requirement for embryonic DA neuron specification in the VNC.

*Δ Notch*			
	St8	St9–10	St10–11
*No. H-ce*lls	% (n = 97)	% (n = 298)	% (n = 569)
1	86.6	25.8	18.3
2	12.4	57.4	71.4
3	0%	12.4	5.8
4	1	3.7	4.6

*No. PM cells*	% (n = 138)	% (n = 238)	% (n = 285)
1	83.3	18.9	28.4
2	14.5	50.4	46.7
3	2.2	28.6	20
4	0%	2.1	4.2

*No. DL ce*lls	% (n = 165)	% (n = 283)	% (n = 120)
1	77	98.9	99.2
2	23	1.1	0.8

Tabular overview of analysis of DA neurons in the VNC after conditional removal of Notch at different phases of development. Presented are the percentages of affected neuromeres or hemineuromeres of H-cells, paramedial (PM) and dorsal lateral (DL) cells when *Notch* function is removed at St8, St9–10 and St10–11 of embryonic development. *Notch* removal during St10–11 of embryogenesis results in a shift towards higher percentage of H-cell duplication and lower percentage of segments containing more than 2 H-cells as compared to when Notch function is removed during St9–10 of embryogenesis, suggesting that sibling cell fate specification involving Notch occurs primarily at around St10–11 for the H-cells.

**Figure 9:**
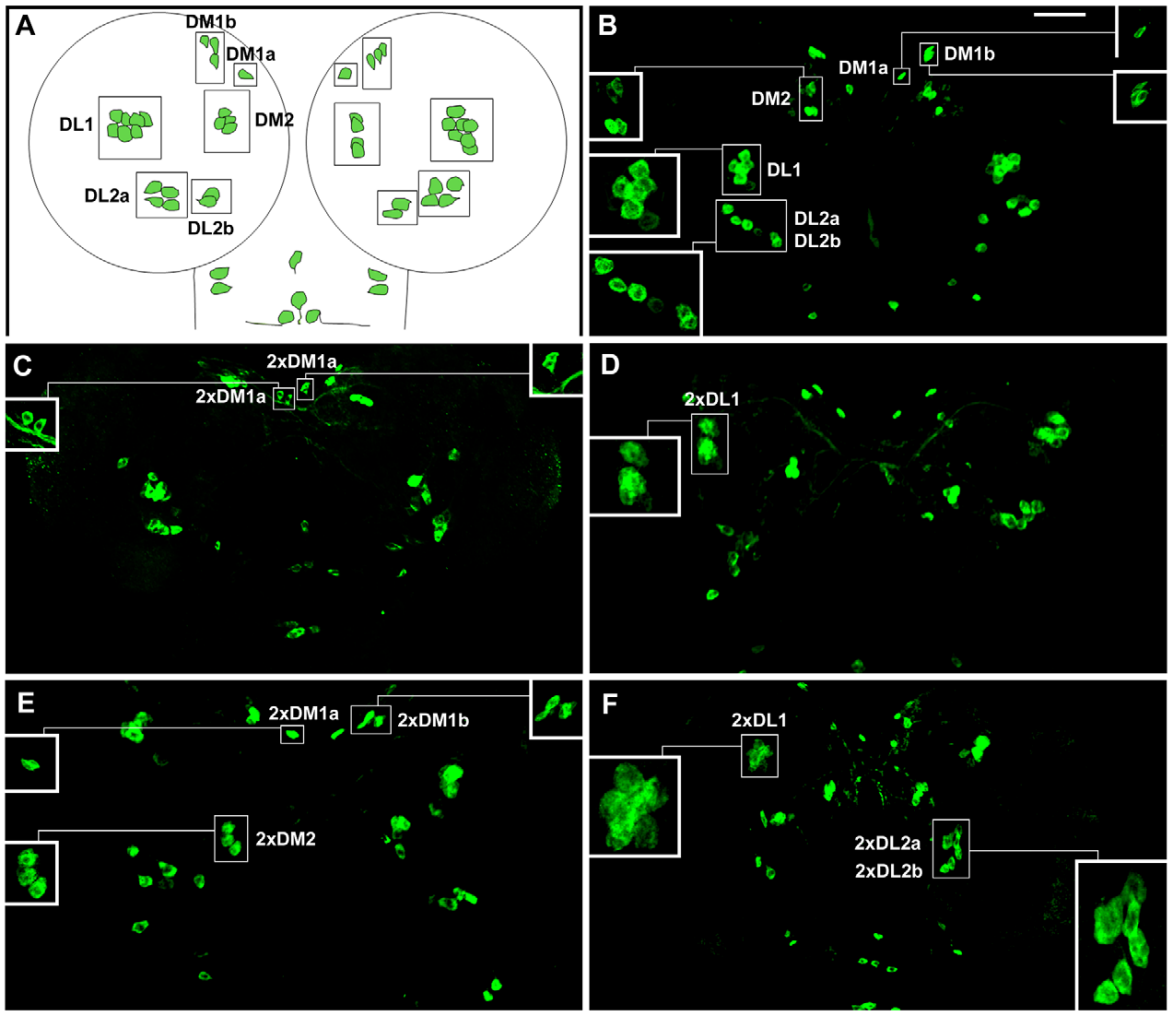
Notch affects specification of DA neurons in the larval brain hemispheres. (A) Schematic diagram of third larval instar brain showing wt neuronal clusters which express TH; the dorsal medial clusters DM1a (1 cell), DM1b (3 cells), DM2 (4 cells), the dorsal lateral clusters DL1 (7cells), DL2a (4 cells) and DL2b (2 cells). (B) Third larval instar brain of *N^ts^* grown at permissive temperature showing normal TH expression. (C–F) Examples of third larval instar brains of *N^ts^*, shifted to restrictive temperature at St9–11, showing duplication of TH-positive neurons at DM1a (both brain hemispheres, C), DL1 (right brain hemisphere, D), DM (both brain hemispheres, E) and DL1 and DL2 (right and left brain hemispheres, respectively, F).

To study Notch function as well as its temporal requirement for DA neuronal specification in the larval hemispheres, we used the conditional *N^ts^* allele to remove *Notch* function for two hours at different developmental stages (i.e. at St9–11, St13 and St16–17 of embryonic development as well as early first larval instar) and analyzed the number of TH expressing cells in third larval instar hemispheres. Removing *Notch* function at St13 onwards till larval stage had very little effect on DA fate specification in the larval hemispheres and only the DM1b clusters was affected with 8.3% of clusters having increased numbers of TH-positive cells (n = 24). However, when *Notch* function was removed during St9–11, DA fate specification was more broadly affected. From the 40 hemispheres investigated, we found a general increase of TH expressing cells in the following clusters: DM1a (17.5% with 2 cells, 15% with more than 2 cells), DM1b (53%>3 cells), DM2 (not affected), DL1 (10%>7 cells), DL2a (5%>4 cells) and DL2b (15%>2 cells) (Fig. 9; Table 4). To confirm that the additional cells indeed represented sibling cell fate transformations, we traced the axonal fasciculation and projection patterns of all TH-expressing cells and found that all cells in these clusters exhibited patterns comparable to the normal cells in these positions (data not shown).

**Table 4:** Temporal analysis of *Notch* requirement for the specification of DA neurons in the larval central brain.

			*Clusters*			
*Δ N*	DM1a	DM1b	DM2	DL1	DL2a	DL2b
St9–11 (n = 40)	17.5% (2 cells), 15% (>2 cells)	53%	0%	10%	5%	15%
St13 (n = 24)	0%	8.3%	0%	0%	0%	0%
St16–17 (n = 54)	0%	0%	0%	0%	0%	0%
1st instar (n = 30)	0%	0%	0%	0%	0%	0%
control (n = 38)	0%	2.6%	0%	0%	0%	0%

Shown are percentages of affected clusters in the larval central brain. Note that only removal of *Notch* function between St9–11 of embryonic development affects the specification of all TH-positive clusters, with the exception of DM2 cluster. Removal of *Notch* function at other stages has no significant effects on numbers of DA neurons in the clusters. This suggests that DA neurons in the hemispheres are born and specified early in embryonic development.

In the larval VNC, increased number of cells were also seen in SM1 (10%), SM2 (32%), TM1 (26%), TM2-A7 (11%) and dorsal lateral cells (2%). Although we cannot rule out the possibility that the phenotypes we observed in the larval central brains were also due to mild neurogenic defects, we suggest that the observed additional cells were caused by defects in binary cell fate specification as the VNCs of the same larva in general did not show detectable neurogenic phenotypes. In addition, the fact that duplication of whole clusters was rarely seen suggests that removal of *Notch* at St9–11 largely affecting binary cell fate choice rather than generation of NBs.

Taken together, our data strongly indicate that Notch signaling controls fate specification of most if not all DA neurons in *Drosophila*.

## Discussion

DA neurons in *Drosophila* have been shown to play roles in behavior [21] as well as learning and memory [51], [52]. While quite recently the roles of asymmetric cell division (ACD) and Notch signaling have been demonstarted for the specification of the ventral midline derived H-cell, not much is known about the mechanisms of specification for the majority of DA neurons which are derived from the lateral and procephalic neuroectoderm. Here, we demonstrate that asymmetric cell division and Notch signaling are both required for the specification of most if not all DA neurons in *Drosophila*. Our data provides further insight into the genesis and mechanisms involved in the specification of DA neurons in general possibly with relevance to vertebrate systems.

### Larval DA neurons are born during embryonic neurogenesis

Our analysis indicates that although the majority of larval DA neurons are fully developed or matured at first larval instar, they are born and specified during embryonic neurogenesis. This is supported by a number of observations: firstly, the majority of DA neurons are already present at early first larval instar, a stage with very limited larval neurogenesis as most neuroblasts are in a state of quiescence [53]. Secondly, larval DA neurons do not express Neurotactin, a specific marker for secondary neurons born during larval neurogenesis. Thirdly, dissection of temporal requirement of Notch clearly shows that Notch is required during embryonic St9–11 for DA neuronal specification (see also below). Strikingly, although DA neurons are born and specified during mid-embryogenesis, the expression of TH protein in most DA neurons can only be detected at late embryonic to early larval stages. The cause of the delay between cell fate specification and neurotransmitter maturation of DA neurons is not understood and will require further analysis. It is possible, however, that mechanisms involving post-translational regulation of TH, e.g. via microRNAs (miRNA) are playing a role in this context as the *Drosophila TH* mRNA contains multiple predicted miRNA binding sites in its 3′ untranslated region [54].

### DA neuronal specification requires asymmetric cell division

Asymmetric cell division (ACD) is a major mechanism for generating cell type diversity in development [1], [55]. However, it has not been shown whether ACD as a general mechanism contributes to the specification of DA neurons in *Drosophila*. Our analysis of asymmetric protein localization patterns in the ventral midline supports previously published result [23]. We found that most MPs localized Insc and Numb asymmetrically suggesting that the intrinsic machinery responsible for cell polarity during cell division is also observed for MPs. Functional analyses of mutations revealed a role for ACD in the specification of DA neurons including those outside of the ventral midline. We found that removal of *insc*, *baz* as well as *numb* and *spdo* affects DA neuronal specification in general. While removal of apical complex proteins generally results in additional DA neurons, the reverse phenotype is observed when basal components are removed. However, in *insc* mutant a less frequent duplication of the dorsal lateral DA neurons in the VNC is observed. Also, specification of the paramedial (PM) DA neurons is unaffected in *insc* mutants. This suggests that Insc may either be partially redundant or may not be required for specification of PM DA neurons. A partial redundancy of Insc has been described for the specification of MP2 cells [56] as well as in late born cells within some NB lineages [49].

Specification of DA neurons in the larval brain hemispheres also requires ACD. *insc*, *numb* and *spdo* mutant clones generated during embryogenesis or *da-GAL4; baz siRNA* knock-down result in altered numbers of DA neurons in the majority of larval DA clusters. In *insc* and *spdo* clones as well as when *baz* is knocked down, DA clusters generally contain more DA neurons whereas in *numb* clones less DA neurons are observed. However, in *insc* and *baz* mutants, we rarely observed duplication of the whole DA clusters. An explanation could be that DA neurons in each cluster are not originated from the same lineage or *insc* and *baz* mutations only affected certain lineages but not others. Alternatively, Insc is only strictly required in cells born early in the NB lineages hence later born DA neurons within the same clusters are not affected resulting in less than double the size of neurons in these clusters. Differential requirement for *insc* based on the birth order of cells in NB lineages has been demonstrated in some embryonic NB lineages [49]. Collectively, our data indicates a critical role of the ACD mechanism in fate specification of most DA neurons in *Drosophila*.

### Notch is required for the specification of DA neurons

We and others [23] have shown that the midline gives rise to one DA neuron per segment called the H-cell which derives from the MP3. Consistent with Wheeler et al. [23], we found approximately 6–7 TH+ cells in the ventral midline of *Notch* mutants. This number of cells is, however, inconsistent with a sole role of Notch in binary sibling cell fate specification of the MP3. It was suggested that Notch is required in differential fate specification of MPs and that in *Notch* additional MPs acquire MP3 cell fate [23]. Thus, the role of Notch could be to repress other MPs to adopt an MP3 fate. Such a repressive role on neighboring cells is similar to the role of Notch in repressing ectodermal cells to take on a NB fate during the process of lateral inhibition [3], [57]. However, not much is known about the differential temporal requirement of Notch for MP specification and binary cell fate specification. Our temporal analysis using a conditional *Notch* allele revealed two possible overlapping phases of Notch requirement in the midline. Removal of Notch during St10–11 generated predominantly two H-cells. This timing is consistent with the MP divisions occurring at late St10 to St11 [23], also stages when binary sibling cell fate specification involving Notch signaling is normally taking place. When Notch function was removed at St9 of embryonic development, 17% of segments were found to contain between 3 to 5 H-cells. We are unable to completely rule out a possible overlap between the requirement for Notch during MP specification and binary sibling cell fate specification, but our data suggests that Notch requirement for MP3 specification begins at St8 and extends into stage 9–10 of embryonic development.

Further to that, we have shown that loss of Notch also results in supernumerary DA neurons outside of the midline. For example, conditional removal of Notch function resulted in additional dorsal lateral and PM DA neurons. Thus, our data reveals a general role of Notch in the specification of DA neurons. Further support for a critical role of Notch in this context comes from our analysis of *numb* and *spdo* mutants. Numb is described as a repressor of Notch [58]. Consequently, we found that *numb* loss of function mutation results in reduced number of DA neurons possibly due to an upregulation of Notch function. *spdo* has been reported to potentiate Notch signaling in the cells that lack the Numb protein [59]. As a result, we found additional DA neurons in clones lacking *spdo* function. Therefore, data obtained from studying Notch and the two important Notch regulators Numb and Spdo clearly disclose an essential role for Notch signaling in the specification of most if not all DA neurons in the fly.

Our study reveals a repressive function of Notch in the specification of DA neurons as the cell with active Notch signaling is normally not specified as DA neuron whereas the cell lacking or repressing Notch signal differentiates into DA neuron. Interestingly, a repressive role for Notch on DA neuronal specification is also observed in the frog spinal cord [60]. It was also reported recently that loss of Notch signaling in the zebrafish led to expansion of cell numbers of DA neurons during development [61]. Previous studies done in *Drosophila* CNS reported a role of Notch on the specification of a subset of DA neurons in the embryo [23], [62]. These reports in conjunction with our data support the notion of Notch as a repressor for DA neuronal specification and at the same time suggest a conserved role of Notch in the specification of DA neurons in flies, vertebrates and arthropods. In conclusion, our data clearly demonstrate that the genesis of DA neurons in the fruit fly requires asymmetric cell division and Notch signaling. Thus, it is very likely that DA neuronal specification in the fly follows a common mechanism requiring the repression of Notch signaling. It also remains to be determined whether Notch represses TH fate directly or indirectly through currently unidentified Notch target genes. The identification of genes which are actively involved in the specification of DA neurons will shed further insights on the general mechanism of DA neuronal specification possibly not limited to the fly system.

## Supporting Information

Figure S1 **Axonal fasciculation and projection patterns of DA neurons in the larval brain hemisphere.** (A–E) show larval brains labeled with GFP (green, from *TH-GAL4 ; UAS-GFP*) and TH (red). (A′–E′) show only TH expression in the same larval brains as (A–E). (A, A′) DM1a and DM1b, (B, B′) DM2, (C, C′) DL1, (D, D′) DL2a, (E, E′) DL2b, each consists of axons that fasciculate together before projecting further. Arrows point to axonal fasciculations and/or projections. (F) Simplified schematic representation of TH-positive clusters in the central brain hemispheres. Axonal projections which are not followed completely are marked by dashed lines.
(TIF)

Figure S2 **Mutation in** ***spdo*** **results in duplication of SML2 and TML1.** Third larval instar VNCs are shown. (A, B and C) represent stacked images of confocal frames showing double labeling of GFP (green, as clonal marker) and TH (red). (A′, B′ and C′) show stacked images of confocal frames focusing on initial axonal projections of the corresponding cells in A, B and C, respectively. (A) *wt* clones showing two SML2s neighboring SM2 (arrows) and (A′) their initial typical axonal projection patterns (arrowheads). (B, B′, C and C′) *spdo^−^* clones showing duplication of SML2 (B, arrows) and TML1 (C, arrows). (B′ and C′) In all cases, the original as well as the duplicated cells initially fasciculate together and then project laterally (arrowheads) suggesting a complete cell fate transformation.
(TIF)

Figure S3 **Disruption of Notch signaling results in gain of embryonic DA neurons and complete loss of Per expression.** Ventral views of St16–17 embryos. (A, C) *wt* and (B, D) *N^55e11^* embryos. (A) In *wt* embryo, H-cells (arrowheads) are present at the ventral midline (indicated by a line) and dorsal lateral DA neurons are present at the dorsal lateral positions (horizontal arrows). (B) In *N^55e11^*embryo, the H-cells at ventral midline (arrowheads) as well as the dorsal lateral DA neurons (horizontal arrows) are multiplied. On average, 6.6±0.2 H cells per neuromere and 2.9±0.2 dorsal lateral DA neurons per hemineuromere are present in the *N^55e11^* embryo. Numbers represent mean ± SEM. (C) In wild type embryo, Per is expressed in both midline and non-midline cells. (D) In *N^55e11^*, Per expression is completely abolished in the CNS. Horizontal arrows in (D) point to the approximate dorsal lateral positions where the DA neurons are missing.
(TIF)

Figure S4 **Gain or loss of Notch affects H-cell specification.** VNCs of third larval instar are shown. (A, D) *wt*, (B, E) *sim-GAL4 ; UAS-N^intra^* and (C, F) *sim-GAL ; UAS-numb*. In the VNC of wild type larva (A, D), one H-cell can be found at the midline (SM1 to AM7). In the VNC of *sim-GAL4 ; UAS-N-intra* larva, H-cells are completely lost from the midline (asterisks and bracketed in B, E) and in the VNC of *sim-GAL4 ; UAS-numb*, the H-cells are duplicated (as marked by 2× in C, F). DLs as well as SML2s and TML1s (arrows in B and C) are not affected by the manipulation of Notch signaling in the midline, indicating that they are not of midline origin.
(TIF)

## References

[1] Horvitz HR, Herskowitz I (1992). Mechanisms of asymmetric cell division: two Bs or not two Bs, that is the question.. Cell.

[2] Matsuzaki F (2000). Asymmetric division of Drosophila neural stem cells: a basis for neural diversity.. Curr Opin Neurobiol.

[3] Cabrera CV (1990). Lateral inhibition and cell fate during neurogenesis in Drosophila: the interactions between scute, Notch and Delta.. Development.

[4] Hartenstein V, Younossi-Hartenstein A, Lekven A (1994). Delamination and division in the Drosophila neurectoderm: spatiotemporal pattern, cytoskeletal dynamics, and common control by neurogenic and segment polarity genes.. Dev Biol.

[5] Kraut R, Campos-Ortega JA (1996). inscuteable, a neural precursor gene of Drosophila, encodes a candidate for a cytoskeleton adaptor protein.. Dev Biol.

[6] Kuchinke U, Grawe F, Knust E (1998). Control of spindle orientation in Drosophila by the Par-3-related PDZ-domain protein Bazooka.. Curr Biol.

[7] Schober M, Schaefer M, Knoblich JA (1999). Bazooka recruits Inscuteable to orient asymmetric cell divisions in Drosophila neuroblasts.. Nature.

[8] Knoblich JA, Jan LY, Jan YN (1997). The N terminus of the Drosophila Numb protein directs membrane association and actin-dependent asymmetric localization.. Proc Natl Acad Sci U S A.

[9] Cayouette M, Raff M (2002). Asymmetric segregation of Numb: a mechanism for neural specification from Drosophila to mammals.. Nat Neurosci.

[10] Lu B, Rothenberg M, Jan LY, Jan YN (1998). Partner of Numb colocalizes with Numb during mitosis and directs Numb asymmetric localization in Drosophila neural and muscle progenitors.. Cell.

[11] Buescher M, Yeo SL, Udolph G, Zavortink M, Yang X (1998). Binary sibling neuronal cell fate decisions in the Drosophila embryonic central nervous system are nonstochastic and require inscuteable-mediated asymmetry of ganglion mother cells.. Genes Dev.

[12] Spana EP, Doe CQ (1996). Numb antagonizes Notch signaling to specify sibling neuron cell fates.. Neuron.

[13] Uemura T, Shepherd S, Ackerman L, Jan LY, Jan YN (1989). numb, a gene required in determination of cell fate during sensory organ formation in Drosophila embryos.. Cell.

[14] Skeath JB, Doe CQ (1998). Sanpodo and Notch act in opposition to Numb to distinguish sibling neuron fates in the Drosophila CNS.. Development.

[15] Dye CA, Lee JK, Atkinson RC, Brewster R, Han PL (1998). The Drosophila sanpodo gene controls sibling cell fate and encodes a tropomodulin homolog, an actin/tropomyosin-associated protein.. Development.

[16] Thomas JB, Crews ST, Goodman CS (1988). Molecular genetics of the single-minded locus: a gene involved in the development of the Drosophila nervous system.. Cell.

[17] Nambu JR, Franks RG, Hu S, Crews ST (1990). The single-minded gene of Drosophila is required for the expression of genes important for the development of CNS midline cells.. Cell.

[18] Wheeler SR, Kearney JB, Guardiola AR, Crews ST (2006). Single-cell mapping of neural and glial gene expression in the developing Drosophila CNS midline cells.. Dev Biol.

[19] Hornykiewicz O (1992). Mechanisms of neuronal loss in Parkinson's disease: a neuroanatomical-biochemical perspective.. Clin Neurol Neurosurg.

[20] German DC, Manaye K, Smith WK, Woodward DJ, Saper CB (1989). Midbrain dopaminergic cell loss in Parkinson's disease: computer visualization.. Ann Neurol.

[21] Neckameyer WS (1998). Dopamine modulates female sexual receptivity in Drosophila melanogaster.. J Neurogenet.

[22] Goridis C, Rohrer H (2002). Specification of catecholaminergic and serotonergic neurons.. Nat Rev Neurosci.

[23] Wheeler SR, Stagg SB, Crews ST (2008). Multiple Notch signaling events control Drosophila CNS midline neurogenesis, gliogenesis and neuronal identity.. Development.

[24] Friggi-Grelin F, Coulom H, Meller M, Gomez D, Hirsh J (2003). Targeted gene expression in Drosophila dopaminergic cells using regulatory sequences from tyrosine hydroxylase.. J Neurobiol.

[25] Xiao H, Hrdlicka LA, Nambu JR (1996). Alternate functions of the single-minded and rhomboid genes in development of the Drosophila ventral neuroectoderm.. Mech Dev.

[26] Wang S, Younger-Shepherd S, Jan LY, Jan YN (1997). Only a subset of the binary cell fate decisions mediated by Numb/Notch signaling in Drosophila sensory organ lineage requires Suppressor of Hairless.. Development.

[27] Struhl G, Fitzgerald K, Greenwald I (1993). Intrinsic activity of the Lin-12 and Notch intracellular domains in vivo.. Cell.

[28] Harrison DA, Perrimon N (1993). Simple and efficient generation of marked clones in Drosophila.. Curr Biol.

[29] Lacin H, Zhu Y, Wilson BA, Skeath JB (2009). dbx mediates neuronal specification and differentiation through cross-repressive, lineage-specific interactions with eve and hb9.. Development.

[30] Bossing T, Udolph G, Doe CQ, Technau GM (1996). The embryonic central nervous system lineages of Drosophila melanogaster. I. Neuroblast lineages derived from the ventral half of the neuroectoderm.. Dev Biol.

[31] Schmidt H, Rickert C, Bossing T, Vef O, Urban J (1997). The embryonic central nervous system lineages of Drosophila melanogaster. II. Neuroblast lineages derived from the dorsal part of the neuroectoderm.. Dev Biol.

[32] Schmid A, Chiba A, Doe CQ (1999). Clonal analysis of Drosophila embryonic neuroblasts: neural cell types, axon projections and muscle targets.. Development.

[33] Lee T, Luo L (2001). Mosaic analysis with a repressible cell marker (MARCM) for Drosophila neural development.. Trends Neurosci.

[34] Lee T, Luo L (1999). Mosaic analysis with a repressible cell marker for studies of gene function in neuronal morphogenesis.. Neuron.

[35] Tio M, Udolph G, Yang X, Chia W (2001). cdc2 links the Drosophila cell cycle and asymmetric division machineries.. Nature.

[36] Yang Y, Gehrke S, Imai Y, Huang Z, Ouyang Y (2006). Mitochondrial pathology and muscle and dopaminergic neuron degeneration caused by inactivation of Drosophila Pink1 is rescued by Parkin.. Proc Natl Acad Sci U S A.

[37] Liu X, Zwiebel LJ, Hinton D, Benzer S, Hall JC (1992). The period gene encodes a predominantly nuclear protein in adult Drosophila.. J Neurosci.

[38] Neckameyer WS, White K (1993). Drosophila tyrosine hydroxylase is encoded by the pale locus.. J Neurogenet.

[39] Lundell MJ, Hirsh J (1994). Temporal and spatial development of serotonin and dopamine neurons in the Drosophila CNS.. Dev Biol.

[40] Selcho M, Pauls D, Han KA, Stocker RF, Thum AS (2009). The role of dopamine in Drosophila larval classical olfactory conditioning.. PLoS One.

[41] Budnik V, White K (1988). Catecholamine-containing neurons in Drosophila melanogaster: distribution and development.. J Comp Neurol.

[42] Cardona A, Saalfeld S, Arganda I, Pereanu W, Schindelin J (2010). Identifying neuronal lineages of Drosophila by sequence analysis of axon tracts.. J Neurosci.

[43] Doe CQ (1992). Molecular markers for identified neuroblasts and ganglion mother cells in the Drosophila central nervous system.. Development.

[44] Broadus J, Skeath JB, Spana EP, Bossing T, Technau G (1995). New neuroblast markers and the origin of the aCC/pCC neurons in the Drosophila central nervous system.. Mech Dev.

[45] Lundell MJ, Lee HK, Perez E, Chadwell L (2003). The regulation of apoptosis by Numb/Notch signaling in the serotonin lineage of Drosophila.. Development.

[46] Ni JQ, Markstein M, Binari R, Pfeiffer B, Liu LP (2008). Vector and parameters for targeted transgenic RNA interference in Drosophila melanogaster.. Nat Methods.

[47] Ni JQ, Liu LP, Binari R, Hardy R, Shim HS (2009). A Drosophila resource of transgenic RNAi lines for neurogenetics.. Genetics.

[48] Udolph G (2010). Notch signaling and the generation of cell diversity in Drosophila neuroblast lineages..

[49] Udolph G, Rath P, Tio M, Toh J, Fang W (2009). On the roles of Notch, Delta, kuzbanian, and inscuteable during the development of Drosophila embryonic neuroblast lineages.. Dev Biol.

[50] Bossing T, Technau GM (1994). The fate of the CNS midline progenitors in Drosophila as revealed by a new method for single cell labelling.. Development.

[51] Zhang S, Yin Y, Lu H, Guo A (2008). Increased dopaminergic signaling impairs aversive olfactory memory retention in Drosophila.. Biochem Biophys Res Commun.

[52] Schwaerzel M, Monastirioti M, Scholz H, Friggi-Grelin F, Birman S (2003). Dopamine and octopamine differentiate between aversive and appetitive olfactory memories in Drosophila.. J Neurosci.

[53] Truman JW, Bate M (1988). Spatial and temporal patterns of neurogenesis in the central nervous system of Drosophila melanogaster.. Dev Biol.

[54] Stark A, Brennecke J, Russell RB, Cohen SM (2003). Identification of Drosophila MicroRNA targets.. PLoS Biol.

[55] Roegiers F, Jan YN (2004). Asymmetric cell division.. Curr Opin Cell Biol.

[56] Rath P, Lin S, Udolph G, Cai Y, Yang X (2002). Inscuteable-independent apicobasally oriented asymmetric divisions in the Drosophila embryonic CNS.. EMBO Rep.

[57] Pan D, Rubin GM (1997). Kuzbanian controls proteolytic processing of Notch and mediates lateral inhibition during Drosophila and vertebrate neurogenesis.. Cell.

[58] Campos-Ortega JA (1996). Numb diverts notch pathway off the tramtrack.. Neuron.

[59] Babaoglan AB, O'Connor-Giles KM, Mistry H, Schickedanz A, Wilson BA (2009). Sanpodo: a context-dependent activator and inhibitor of Notch signaling during asymmetric divisions.. Development.

[60] Binor E, Heathcote RD (2005). Activated notch disrupts the initial patterning of dopaminergic spinal cord neurons.. Dev Neurosci.

[61] Mahler J, Filippi A, Driever W (2010). DeltaA/DeltaD regulate multiple and temporally distinct phases of notch signaling during dopaminergic neurogenesis in zebrafish.. J Neurosci.

[62] Stagg SB, Guardiola AR, Crews ST (2011). Dual role for Drosophila lethal of scute in CNS midline precursor formation and dopaminergic neuron and motoneuron cell fate.. Development.

