# Supplementary figures and images for: Asymmetric Cell Division and Notch Signaling Specify Dopaminergic Neurons in Drosophila
Source: PLoS One. 2011 Nov 4;6(11):e26879. doi: 10.1371/journal.pone.0026879 (PMC3208554; doi:10.1371/journal.pone.0026879)

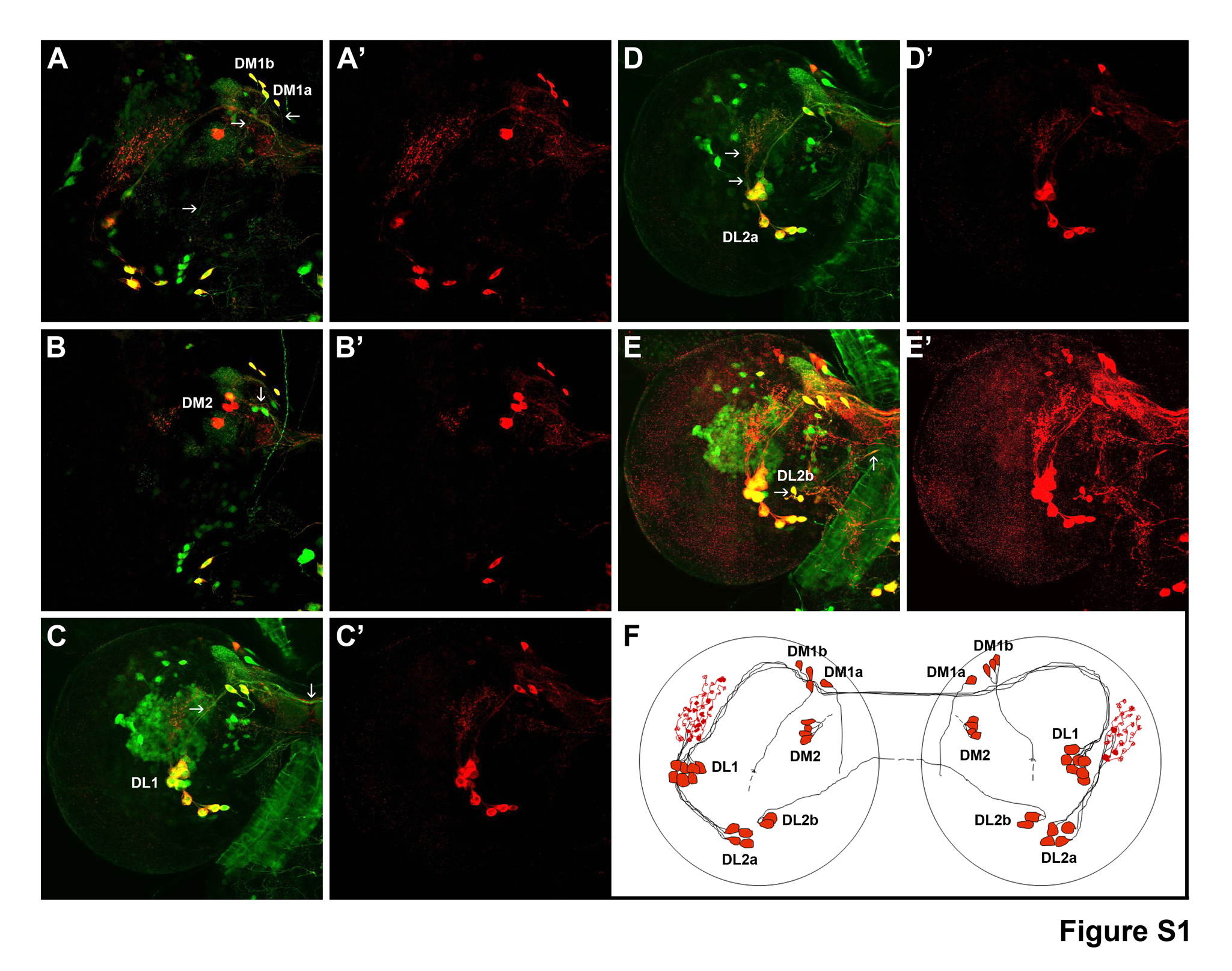

Supplement: Figure S1 — Axonal fasciculation and projection patterns of DA neurons in the larval brain hemisphere. (A–E) show larval brains labeled with GFP (green, from TH-GAL4 ; UAS-GFP) and TH (red). (A′–E′) show only TH expression in the same larval brains as (A–E). (A, A′) DM1a and DM1b, (B, B′) DM2, (C, C′) DL1, (D, D′) DL2a, (E, E′) DL2b, each consists of axons that fasciculate together before projecting further. Arrows point to axonal fasciculations and/or projections. (F) Simplified schematic representation of TH-positive clusters in the central brain hemispheres. Axonal projections which are not followed completely are marked by dashed lines. (TIF) [file pone.0026879.s001.tif]

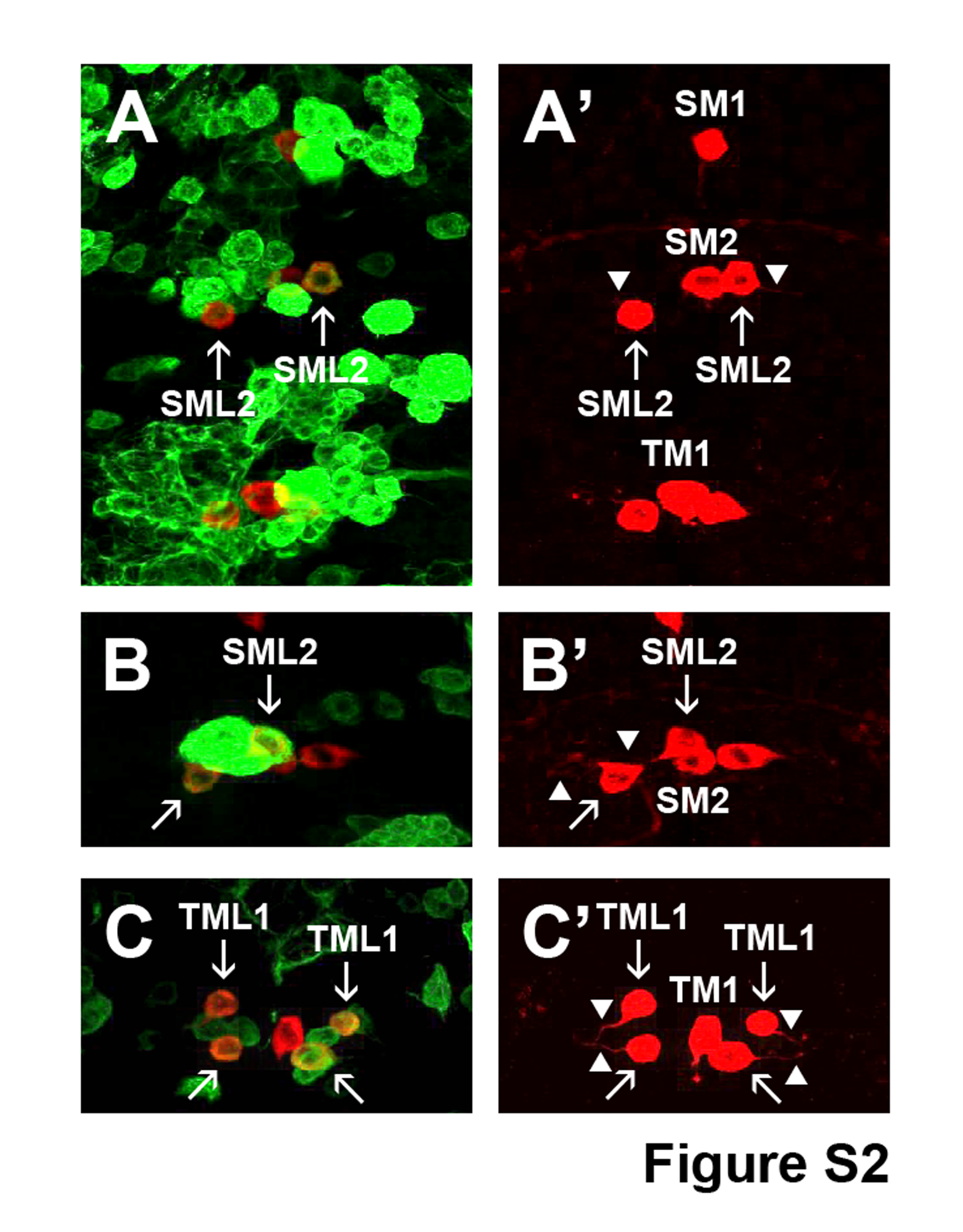

Supplement: Figure S2 — Mutation in spdo results in duplication of SML2 and TML1. Third larval instar VNCs are shown. (A, B and C) represent stacked images of confocal frames showing double labeling of GFP (green, as clonal marker) and TH (red). (A′, B′ and C′) show stacked images of confocal frames focusing on initial axonal projections of the corresponding cells in A, B and C, respectively. (A) wt clones showing two SML2s neighboring SM2 (arrows) and (A′) their initial typical axonal projection patterns (arrowheads). (B, B′, C and C′) spdo− clones showing duplication of SML2 (B, arrows) and TML1 (C, arrows). (B′ and C′) In all cases, the original as well as the duplicated cells initially fasciculate together and then project laterally (arrowheads) suggesting a complete cell fate transformation. (TIF) [file pone.0026879.s002.tif]

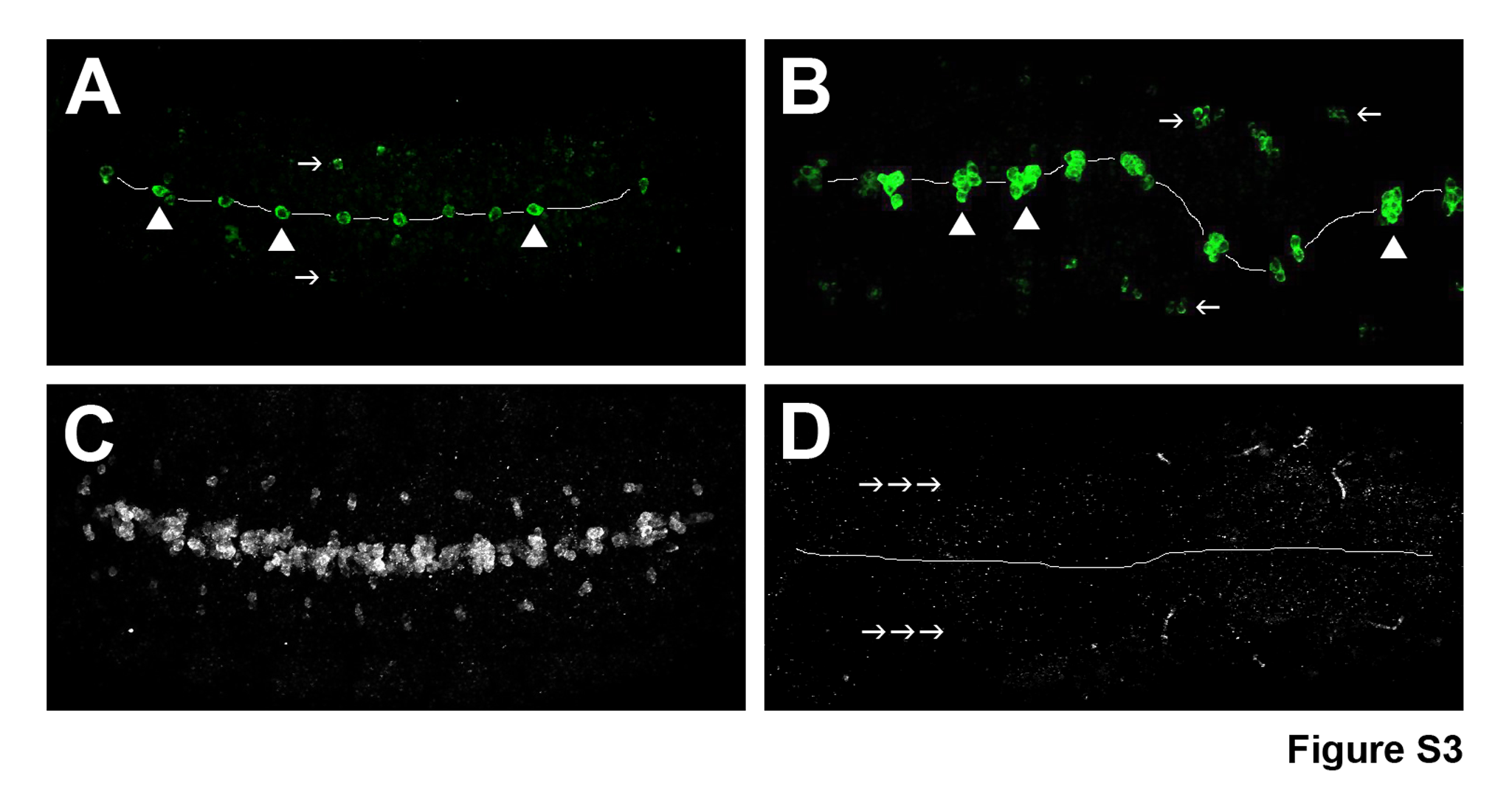

Supplement: Figure S3 — Disruption of Notch signaling results in gain of embryonic DA neurons and complete loss of Per expression. Ventral views of St16–17 embryos. (A, C) wt and (B, D) N55e11 embryos. (A) In wt embryo, H-cells (arrowheads) are present at the ventral midline (indicated by a line) and dorsal lateral DA neurons are present at the dorsal lateral positions (horizontal arrows). (B) In N55e11embryo, the H-cells at ventral midline (arrowheads) as well as the dorsal lateral DA neurons (horizontal arrows) are multiplied. On average, 6.6±0.2 H cells per neuromere and 2.9±0.2 dorsal lateral DA neurons per hemineuromere are present in the N55e11 embryo. Numbers represent mean ± SEM. (C) In wild type embryo, Per is expressed in both midline and non-midline cells. (D) In N55e11, Per expression is completely abolished in the CNS. Horizontal arrows in (D) point to the approximate dorsal lateral positions where the DA neurons are missing. (TIF) [file pone.0026879.s003.tif]

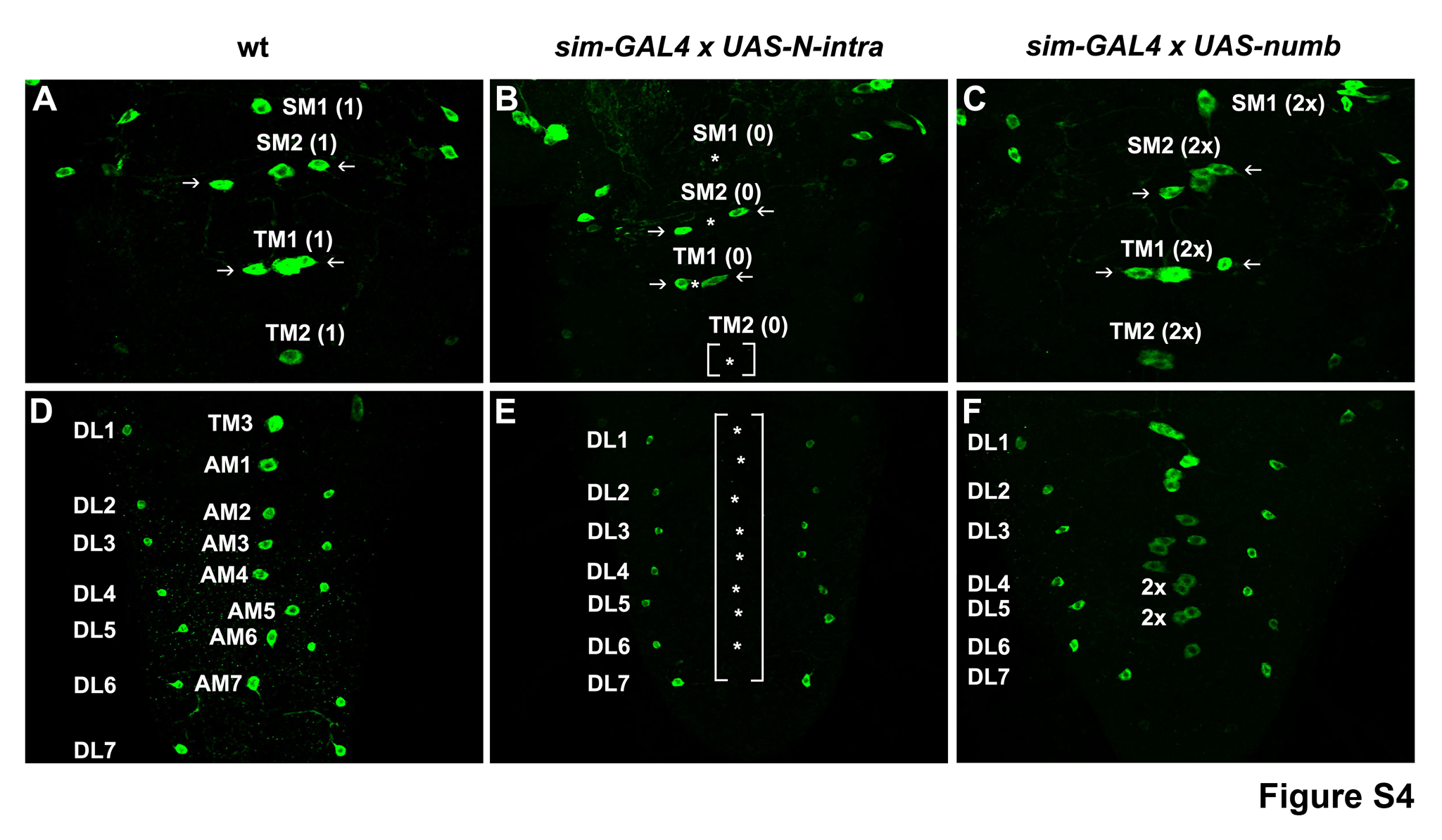

Supplement: Figure S4 — Gain or loss of Notch affects H-cell specification. VNCs of third larval instar are shown. (A, D) wt, (B, E) sim-GAL4 ; UAS-Nintra and (C, F) sim-GAL ; UAS-numb. In the VNC of wild type larva (A, D), one H-cell can be found at the midline (SM1 to AM7). In the VNC of sim-GAL4 ; UAS-N-intra larva, H-cells are completely lost from the midline (asterisks and bracketed in B, E) and in the VNC of sim-GAL4 ; UAS-numb, the H-cells are duplicated (as marked by 2× in C, F). DLs as well as SML2s and TML1s (arrows in B and C) are not affected by the manipulation of Notch signaling in the midline, indicating that they are not of midline origin. (TIF) [file pone.0026879.s004.tif]
